# Crystal structure of 3-benzyl-1-[(cyclo­hexyl­idene)amino]­thio­urea

**DOI:** 10.1107/S205698901502112X

**Published:** 2015-11-14

**Authors:** Shaaban K. Mohamed, Joel T. Mague, Mehmet Akkurt, Alaa A. Hassan, Ahmed T. Abdel-Aziz, Mustafa R. Albayati

**Affiliations:** a Faculty of Science & Engineering, School of Healthcare Science, Manchester Metropolitan University, Manchester M1 5GD, England; b Chemistry Department, Faculty of Science, Minia University, 61519 El-Minia, Egypt; c Department of Chemistry, Tulane University, New Orleans, LA 70118, USA; d Department of Physics, Faculty of Sciences, Erciyes University, 38039 Kayseri, Turkey; e Kirkuk University, College of Education, Department of Chemistry, Kirkuk, Iraq

**Keywords:** crystal structure, thio­ureas, chelating agents, hydrogen bonding

## Abstract

The conformation of the title compound, C_14_H_19_N_3_S, is partially determined by an intra­molecular N—H⋯N hydro­gen-bond inter­action, although the N—H⋯N angle of 108° is quite small. The cyclo­hexyl­idene ring has a chair conformation and its mean plane is inclined to the benzene ring by 46.30 (8)°. In the crystal, mol­ecules are linked by pairs of N—H⋯S hydrogen bonds, forming inversion dimers, with an *R*_2_^2^(8) ring motif. The dimers are reinforced by pairs of C—H⋯S hydrogen bonds, and are linked by further weak C—H⋯S hydrogen bonds, forming chains propagating along [100].

## Related literature   

For pharmacuetical properties of both thio­semicarbazones and their metal complexes, see: Kalinowski & Richardson (2005 ▸, 2007 ▸); Smee & Sidwell (2003 ▸); Pandeya *et al.* (1999 ▸); Beraldo & Gambino (2004 ▸); Chohan *et al.* (2004 ▸). For the synthesis of the title compound, see: Mague *et al.* (2014 ▸).
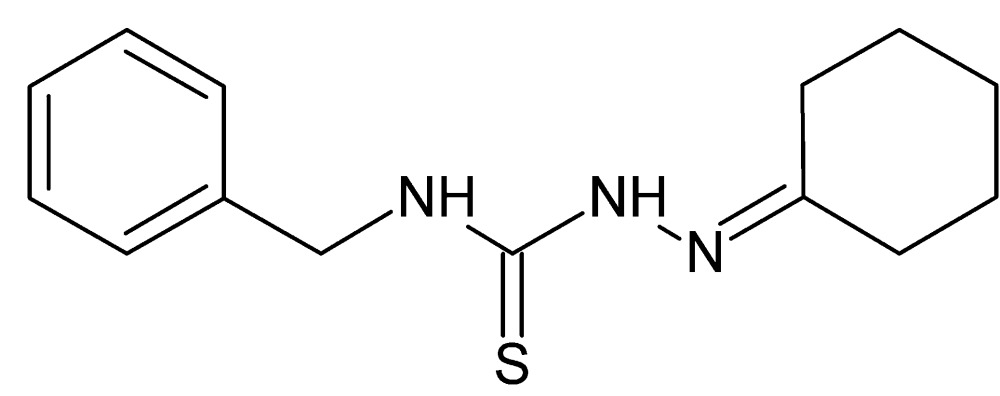


## Experimental   

### Crystal data  

C_14_H_19_N_3_S
*M* *_r_* = 261.38
Triclinic, 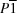

*a* = 6.5537 (3) Å
*b* = 10.5247 (5) Å
*c* = 11.3403 (5) Å
α = 113.682 (1)°
β = 92.969 (2)°
γ = 106.610 (2)°
*V* = 673.96 (5) Å^3^
*Z* = 2
Cu *K*α radiation
μ = 2.01 mm^−1^
*T* = 150 K
0.31 × 0.20 × 0.16 mm

### Data collection  

Bruker D8 VENTURE PHOTON 100 CMOS diffractometer
Absorption correction: multi-scan (*SADABS*; Bruker, 2014 ▸) *T*_min_ = 0.66, *T*_max_ = 0.73
5045 measured reflections
2509 independent reflections
2403 reflections with *I* > 2σ(*I*)
*R*_int_ = 0.019

### Refinement  

*R*[*F* ^2^ > 2σ(*F* ^2^)] = 0.034
*wR*(*F* ^2^) = 0.086
*S* = 1.09
2509 reflections
163 parameters
H-atom parameters constrained
Δρ_max_ = 0.23 e Å^−3^
Δρ_min_ = −0.23 e Å^−3^

### 

Data collection: *APEX2* (Bruker, 2014 ▸); cell refinement: *SAINT* (Bruker, 2014 ▸); data reduction: *SAINT*; program(s) used to solve structure: *SHELXT* (Sheldrick, 2015*a* ▸); program(s) used to refine structure: *SHELXL2014* (Sheldrick, 2015*b* ▸); molecular graphics: *DIAMOND* (Brandenburg & Putz, 2012 ▸); software used to prepare material for publication: *SHELXTL* (Sheldrick, 2008 ▸).

## Supplementary Material

Crystal structure: contains datablock(s) global, I. DOI: 10.1107/S205698901502112X/su5234sup1.cif

Structure factors: contains datablock(s) I. DOI: 10.1107/S205698901502112X/su5234Isup2.hkl

Supporting information file. DOI: 10.1107/S205698901502112X/su5234Isup3.cml

**Fig. 1.**
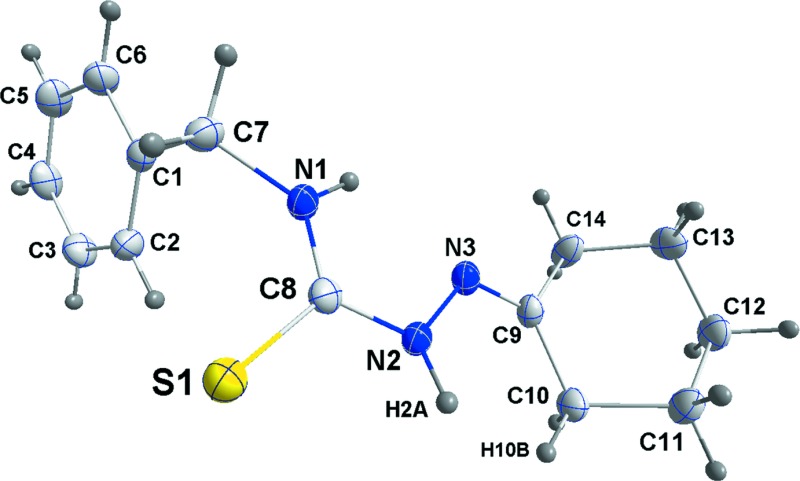
The molecular structure of the title compound, showing the atom-labeling scheme and 50% probability displacement ellipsoids.

**Fig. 2.**
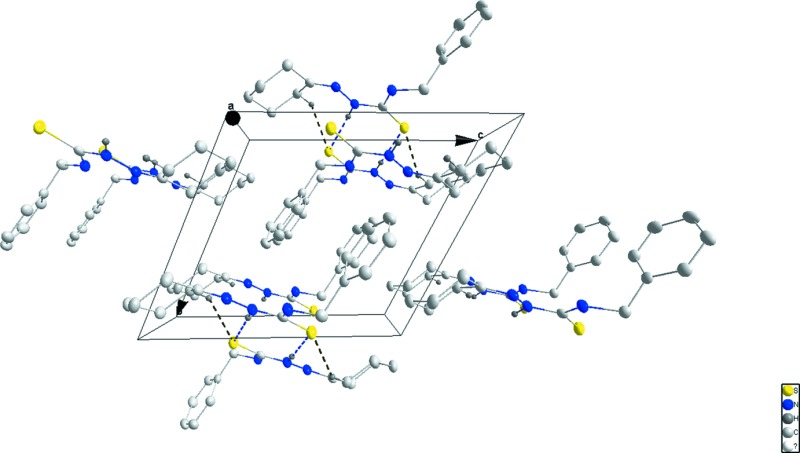
The crystal packing of the title compound, viewed along the a axis. The N—H···S and C—H···S hydrogen bonds appear as dotted lines (see Table 1).

. DOI: 10.1107/S205698901502112X/su5234fig1.tif
The mol­ecular structure of the title compound, showing the atom-labeling scheme and 50% probability displacement ellipsoids.

a . DOI: 10.1107/S205698901502112X/su5234fig2.tif
The crystal packing of the title compound, viewed along the *a* axis. The N—H⋯S and C—H⋯S hydrogen bonds appear as dotted lines (see Table 1).

CCDC reference: 1435497

Additional supporting information:  crystallographic information; 3D view; checkCIF report

## Figures and Tables

**Table 1:** Hydrogen-bond geometry (Å, °)

*D*—H⋯*A*	*D*—H	H⋯*A*	*D*⋯*A*	*D*—H⋯*A*
N1—H1*A*⋯N3	0.91	2.14	2.5713 (17)	108
N2—H2*A*⋯S1^i^	0.91	2.55	3.4577 (12)	172
C10—H10*B*⋯S1^i^	0.99	2.61	3.4847 (14)	147
C7—H7*A*⋯S1^ii^	0.99	2.85	3.8413 (15)	175

Symmetry codes: (i) 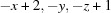; (ii) 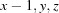.

## supplementary crystallographic information

### S1. Comment

Both thiosemicarbazones and their metal complexes have been studied as potential antiviral, antibacterial, antimycobacterial, antiprotozoal, antifungal, and antineoplastic agents (Kalinowski & Richardson, 2005; Kalinowski & Richardson, 2007; Smee & Sidwell, 2003; Pandeya *et al.*, 1999). Furthermore, their anticonvulsant and neurotropic effects also have been reported (Beraldo & Gambino, 2004). The antifungal properties of thiosemicarbazones can be increased upon complexation with metal ions (Chohan *et al.*, 2004). Based on such facts and following to our on-going study on synthesis of bio-active molecules we report in this study the synthesis and crystal structure of the title compound.

In the title compound, Fig. 1, the cyclohexylidene ring has a chair conformation with puckering parameters of Q = 0.564 (2) Å, θ = 177.2 (2)° and φ = 64 (4)°. The molecular conformation of the molecule may also be partially determined by an intramolecular N1—H1A···N3 hydrogen bond (H1A···N3 = 2.14 Å), although the N1—H1A···N3 angle of 108 ° is quite small (see Table 1).

In the crystal, molecules form inversion dimers through complementary N2—H2A···S1^i^ and C10—H10B···S1^i^ hydrogen bonds (Table 1 and Fig. 2). The dimers are linked by further C-H···S hydrogen bonds forming chains along direction [100]; Table 1.

### S2. Experimental

The title compound was prepared according to our recently reported method (Mague *et al.*, 2014). Colourless crystals suitable for X-ray analysis were obtained by crystallization of the crude product from ethanol (yield 91%; m.p. 375–376 K).

### S3. Refinement

H atoms attached to carbon were placed in calculated positions (C—H = 0.95 - 0.99 Å) while those attached to nitrogen were placed in locations derived from a difference map and their parameters adjusted to give N—H = 0.91 Å. They were all included as riding contributions with U_iso_(H) = 1.2U_eq_(N,C).

### Crystal data

**Table:**

C_14_H_19_N_3_S	*Z* = 2
*M**_r_* = 261.38	*F*(000) = 280
Triclinic, *P*1	*D*_x_ = 1.288 Mg m^−^^3^
*a* = 6.5537 (3) Å	Cu *K*α radiation, λ = 1.54178 Å
*b* = 10.5247 (5) Å	Cell parameters from 4705 reflections
*c* = 11.3403 (5) Å	θ = 4.3–72.1°
α = 113.682 (1)°	µ = 2.01 mm^−^^1^
β = 92.969 (2)°	*T* = 150 K
γ = 106.610 (2)°	Block, colourless
*V* = 673.96 (5) Å^3^	0.31 × 0.20 × 0.16 mm

### Data collection

**Table:**

Bruker D8 VENTURE PHOTON 100 CMOS diffractometer	2509 independent reflections
Radiation source: INCOATEC IµS micro–focus source	2403 reflections with *I* > 2σ(*I*)
Mirror monochromator	*R*_int_ = 0.019
Detector resolution: 10.4167 pixels mm^-1^	θ_max_ = 72.1°, θ_min_ = 4.3°
ω scans	*h* = −7→8
Absorption correction: multi-scan (*SADABS*; Bruker, 2014)	*k* = −12→12
*T*_min_ = 0.66, *T*_max_ = 0.73	*l* = −13→13
5045 measured reflections	

### Refinement

**Table:**

Refinement on *F*^2^	Primary atom site location: structure-invariant direct methods
Least-squares matrix: full	Secondary atom site location: difference Fourier map
*R*[*F*^2^ > 2σ(*F*^2^)] = 0.034	Hydrogen site location: mixed
*wR*(*F*^2^) = 0.086	H-atom parameters constrained
*S* = 1.09	*w* = 1/[σ^2^(*F*_o_^2^) + (0.0345*P*)^2^ + 0.3099*P*] where *P* = (*F*_o_^2^ + 2*F*_c_^2^)/3
2509 reflections	(Δ/σ)_max_ < 0.001
163 parameters	Δρ_max_ = 0.23 e Å^−^^3^
0 restraints	Δρ_min_ = −0.23 e Å^−^^3^

### Special details

**Table:**

Geometry. All e.s.d.'s (except the e.s.d. in the dihedral angle between two l.s. planes) are estimated using the full covariance matrix. The cell e.s.d.'s are taken into account individually in the estimation of e.s.d.'s in distances, angles and torsion angles; correlations between e.s.d.'s in cell parameters are only used when they are defined by crystal symmetry. An approximate (isotropic) treatment of cell e.s.d.'s is used for estimating e.s.d.'s involving l.s. planes.
Refinement. Refinement of *F*^2^ against ALL reflections. The weighted *R*-factor *wR* and goodness of fit *S* are based on *F*^2^, conventional *R*-factors *R* are based on *F*, with *F* set to zero for negative *F*^2^. The threshold expression of *F*^2^ > σ(*F*^2^) is used only for calculating *R*-factors(gt) *etc*. and is not relevant to the choice of reflections for refinement. *R*-factors based on *F*^2^ are statistically about twice as large as those based on *F*, and *R*- factors based on ALL data will be even larger. H-atoms attached to carbon were placed in calculated positions (C—H = 0.95 - 0.99 Å) while those attached to nitrogen were placed in locations derived from a difference map and their parameters adjusted to give N—H = 0.91 Å. All were included as riding contributions with isotropic displacement parameters 1.2 - 1.5 times those of the attached atoms.

### Fractional atomic coordinates and isotropic or equivalent isotropic displacement parameters (Å^2^)

**Table:**

	*x*	*y*	*z*	*U*_iso_*/*U*_eq_	
S1	0.84373 (6)	0.04432 (4)	0.36001 (3)	0.02549 (12)	
N1	0.58096 (18)	0.18119 (13)	0.48809 (11)	0.0220 (3)	
H1A	0.5393	0.2255	0.5652	0.026*	
N2	0.83691 (18)	0.15744 (12)	0.61404 (11)	0.0206 (2)	
H2A	0.9318	0.1107	0.6194	0.025*	
N3	0.73740 (19)	0.21975 (13)	0.71731 (11)	0.0226 (3)	
C1	0.5199 (2)	0.30572 (15)	0.35057 (12)	0.0199 (3)	
C2	0.7361 (2)	0.39067 (16)	0.37044 (13)	0.0242 (3)	
H2	0.8478	0.3640	0.4008	0.029*	
C3	0.7893 (3)	0.51413 (17)	0.34603 (15)	0.0288 (3)	
H3	0.9371	0.5720	0.3603	0.035*	
C4	0.6271 (3)	0.55336 (17)	0.30082 (15)	0.0299 (3)	
H4	0.6637	0.6373	0.2833	0.036*	
C5	0.4118 (3)	0.46964 (17)	0.28133 (15)	0.0292 (3)	
H5	0.3004	0.4962	0.2504	0.035*	
C6	0.3582 (2)	0.34677 (16)	0.30689 (14)	0.0244 (3)	
H6	0.2101	0.2904	0.2944	0.029*	
C7	0.4581 (2)	0.16627 (15)	0.36986 (13)	0.0227 (3)	
H7A	0.3018	0.1364	0.3735	0.027*	
H7B	0.4804	0.0867	0.2930	0.027*	
C8	0.7447 (2)	0.13093 (14)	0.49275 (13)	0.0192 (3)	
C9	0.8216 (2)	0.25262 (15)	0.83473 (13)	0.0228 (3)	
C10	1.0256 (2)	0.23519 (16)	0.88270 (13)	0.0238 (3)	
H10A	1.1294	0.3329	0.9438	0.029*	
H10B	1.0940	0.1919	0.8075	0.029*	
C11	0.9736 (2)	0.13508 (17)	0.95292 (14)	0.0265 (3)	
H11A	0.8894	0.0332	0.8882	0.032*	
H11B	1.1105	0.1339	0.9930	0.032*	
C12	0.8435 (2)	0.18855 (17)	1.05953 (14)	0.0281 (3)	
H12A	0.8036	0.1182	1.0987	0.034*	
H12B	0.9349	0.2853	1.1297	0.034*	
C13	0.6383 (2)	0.20312 (18)	1.00518 (14)	0.0284 (3)	
H13A	0.5616	0.2417	1.0773	0.034*	
H13B	0.5406	0.1050	0.9405	0.034*	
C14	0.6939 (3)	0.30723 (18)	0.93927 (15)	0.0297 (3)	
H14A	0.5589	0.3111	0.8997	0.036*	
H14B	0.7803	0.4079	1.0054	0.036*	

### Atomic displacement parameters (Å^2^)

**Table:**

	*U*^11^	*U*^22^	*U*^33^	*U*^12^	*U*^13^	*U*^23^
S1	0.0279 (2)	0.0337 (2)	0.02208 (19)	0.01730 (16)	0.00831 (13)	0.01400 (15)
N1	0.0244 (6)	0.0269 (6)	0.0220 (6)	0.0131 (5)	0.0072 (4)	0.0143 (5)
N2	0.0229 (6)	0.0239 (6)	0.0212 (6)	0.0113 (5)	0.0064 (4)	0.0131 (5)
N3	0.0276 (6)	0.0239 (6)	0.0229 (6)	0.0123 (5)	0.0088 (5)	0.0135 (5)
C1	0.0234 (7)	0.0219 (7)	0.0152 (6)	0.0089 (6)	0.0042 (5)	0.0079 (5)
C2	0.0228 (7)	0.0274 (7)	0.0234 (7)	0.0084 (6)	0.0031 (5)	0.0123 (6)
C3	0.0291 (7)	0.0267 (8)	0.0282 (7)	0.0043 (6)	0.0071 (6)	0.0129 (6)
C4	0.0425 (9)	0.0251 (7)	0.0286 (7)	0.0132 (7)	0.0121 (6)	0.0161 (6)
C5	0.0355 (8)	0.0308 (8)	0.0304 (8)	0.0182 (7)	0.0075 (6)	0.0172 (6)
C6	0.0241 (7)	0.0275 (7)	0.0249 (7)	0.0113 (6)	0.0054 (5)	0.0126 (6)
C7	0.0197 (6)	0.0241 (7)	0.0253 (7)	0.0067 (6)	0.0006 (5)	0.0125 (6)
C8	0.0188 (6)	0.0177 (6)	0.0231 (6)	0.0044 (5)	0.0045 (5)	0.0119 (5)
C9	0.0291 (7)	0.0214 (7)	0.0234 (7)	0.0108 (6)	0.0070 (5)	0.0130 (6)
C10	0.0250 (7)	0.0258 (7)	0.0209 (6)	0.0085 (6)	0.0043 (5)	0.0103 (6)
C11	0.0314 (7)	0.0296 (8)	0.0241 (7)	0.0150 (6)	0.0047 (6)	0.0139 (6)
C12	0.0339 (8)	0.0333 (8)	0.0225 (7)	0.0131 (7)	0.0067 (6)	0.0159 (6)
C13	0.0282 (7)	0.0366 (8)	0.0216 (7)	0.0122 (6)	0.0086 (6)	0.0126 (6)
C14	0.0369 (8)	0.0361 (8)	0.0250 (7)	0.0227 (7)	0.0099 (6)	0.0144 (6)

### Geometric parameters (Å, º)

**Table:**

S1—C8	1.6898 (13)	C6—H6	0.9500
N1—C8	1.3323 (17)	C7—H7A	0.9900
N1—C7	1.4551 (17)	C7—H7B	0.9900
N1—H1A	0.9098	C9—C10	1.5041 (19)
N2—C8	1.3580 (17)	C9—C14	1.5053 (19)
N2—N3	1.3905 (16)	C10—C11	1.5353 (19)
N2—H2A	0.9098	C10—H10A	0.9900
N3—C9	1.2814 (18)	C10—H10B	0.9900
C1—C6	1.3911 (19)	C11—C12	1.530 (2)
C1—C2	1.392 (2)	C11—H11A	0.9900
C1—C7	1.5144 (18)	C11—H11B	0.9900
C2—C3	1.388 (2)	C12—C13	1.523 (2)
C2—H2	0.9500	C12—H12A	0.9900
C3—C4	1.389 (2)	C12—H12B	0.9900
C3—H3	0.9500	C13—C14	1.533 (2)
C4—C5	1.384 (2)	C13—H13A	0.9900
C4—H4	0.9500	C13—H13B	0.9900
C5—C6	1.390 (2)	C14—H14A	0.9900
C5—H5	0.9500	C14—H14B	0.9900
			
C8—N1—C7	125.66 (12)	N3—C9—C10	128.89 (13)
C8—N1—H1A	117.0	N3—C9—C14	116.47 (12)
C7—N1—H1A	117.3	C10—C9—C14	114.51 (12)
C8—N2—N3	116.90 (11)	C9—C10—C11	110.25 (12)
C8—N2—H2A	117.8	C9—C10—H10A	109.6
N3—N2—H2A	122.8	C11—C10—H10A	109.6
C9—N3—N2	119.80 (12)	C9—C10—H10B	109.6
C6—C1—C2	119.17 (13)	C11—C10—H10B	109.6
C6—C1—C7	119.45 (12)	H10A—C10—H10B	108.1
C2—C1—C7	121.32 (12)	C12—C11—C10	111.12 (12)
C3—C2—C1	120.32 (13)	C12—C11—H11A	109.4
C3—C2—H2	119.8	C10—C11—H11A	109.4
C1—C2—H2	119.8	C12—C11—H11B	109.4
C2—C3—C4	120.19 (14)	C10—C11—H11B	109.4
C2—C3—H3	119.9	H11A—C11—H11B	108.0
C4—C3—H3	119.9	C13—C12—C11	111.75 (12)
C5—C4—C3	119.75 (13)	C13—C12—H12A	109.3
C5—C4—H4	120.1	C11—C12—H12A	109.3
C3—C4—H4	120.1	C13—C12—H12B	109.3
C4—C5—C6	120.13 (14)	C11—C12—H12B	109.3
C4—C5—H5	119.9	H12A—C12—H12B	107.9
C6—C5—H5	119.9	C12—C13—C14	110.59 (12)
C5—C6—C1	120.42 (14)	C12—C13—H13A	109.5
C5—C6—H6	119.8	C14—C13—H13A	109.5
C1—C6—H6	119.8	C12—C13—H13B	109.5
N1—C7—C1	113.77 (11)	C14—C13—H13B	109.5
N1—C7—H7A	108.8	H13A—C13—H13B	108.1
C1—C7—H7A	108.8	C9—C14—C13	109.31 (12)
N1—C7—H7B	108.8	C9—C14—H14A	109.8
C1—C7—H7B	108.8	C13—C14—H14A	109.8
H7A—C7—H7B	107.7	C9—C14—H14B	109.8
N1—C8—N2	115.81 (12)	C13—C14—H14B	109.8
N1—C8—S1	123.98 (10)	H14A—C14—H14B	108.3
N2—C8—S1	120.18 (10)		
			
C8—N2—N3—C9	−177.30 (12)	C7—N1—C8—S1	1.96 (19)
C6—C1—C2—C3	−0.4 (2)	N3—N2—C8—N1	6.42 (17)
C7—C1—C2—C3	176.86 (13)	N3—N2—C8—S1	−175.61 (9)
C1—C2—C3—C4	−0.4 (2)	N2—N3—C9—C10	0.8 (2)
C2—C3—C4—C5	0.6 (2)	N2—N3—C9—C14	−174.84 (12)
C3—C4—C5—C6	0.0 (2)	N3—C9—C10—C11	−120.72 (16)
C4—C5—C6—C1	−0.8 (2)	C14—C9—C10—C11	54.96 (16)
C2—C1—C6—C5	1.0 (2)	C9—C10—C11—C12	−52.49 (16)
C7—C1—C6—C5	−176.31 (13)	C10—C11—C12—C13	55.00 (17)
C8—N1—C7—C1	−102.88 (15)	C11—C12—C13—C14	−56.90 (17)
C6—C1—C7—N1	−138.48 (13)	N3—C9—C14—C13	119.63 (14)
C2—C1—C7—N1	44.22 (17)	C10—C9—C14—C13	−56.62 (17)
C7—N1—C8—N2	179.85 (12)	C12—C13—C14—C9	56.04 (16)

### Hydrogen-bond geometry (Å, º)

**Table:**

*D*—H···*A*	*D*—H	H···*A*	*D*···*A*	*D*—H···*A*
N1—H1*A*···N3	0.91	2.14	2.5713 (17)	108
N2—H2*A*···S1^i^	0.91	2.55	3.4577 (12)	172
C10—H10*B*···S1^i^	0.99	2.61	3.4847 (14)	147
C7—H7*A*···S1^ii^	0.99	2.85	3.8413 (15)	175

Symmetry codes: (i) −*x*+2, −*y*, −*z*+1; (ii) *x*−1, *y*, *z*.
