# Supplementary figures and images for: Crystal structure of 3-benzyl-1-[(cyclo­hexyl­idene)amino]­thio­urea
Source: Acta Crystallogr E Crystallogr Commun. 2015 Nov 14;71(Pt 12):o933–4. doi: 10.1107/S205698901502112X (PMC4719888; doi:10.1107/S205698901502112X)

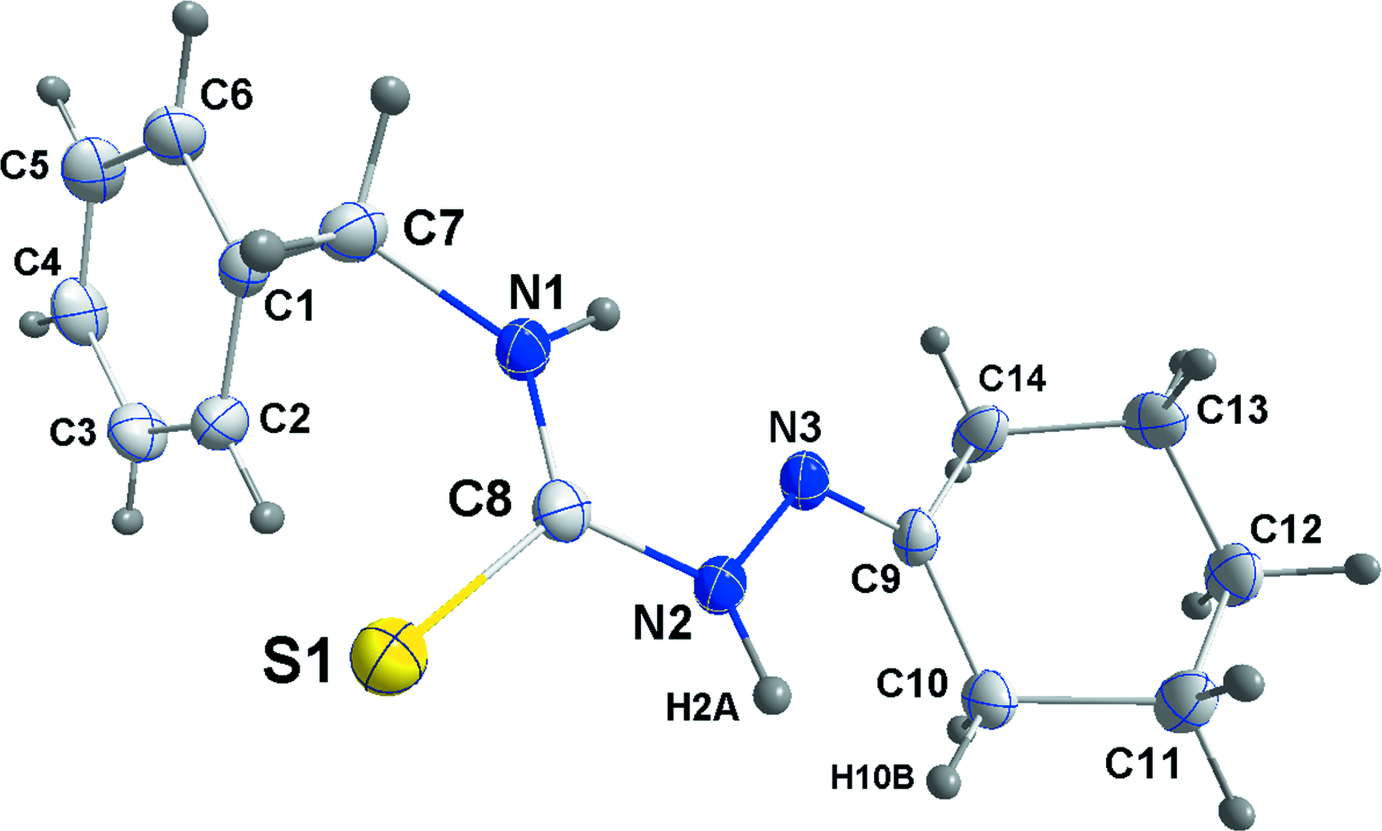

Supplement: Supplementary file 4 [file e-71-0o933-fig1.tif]

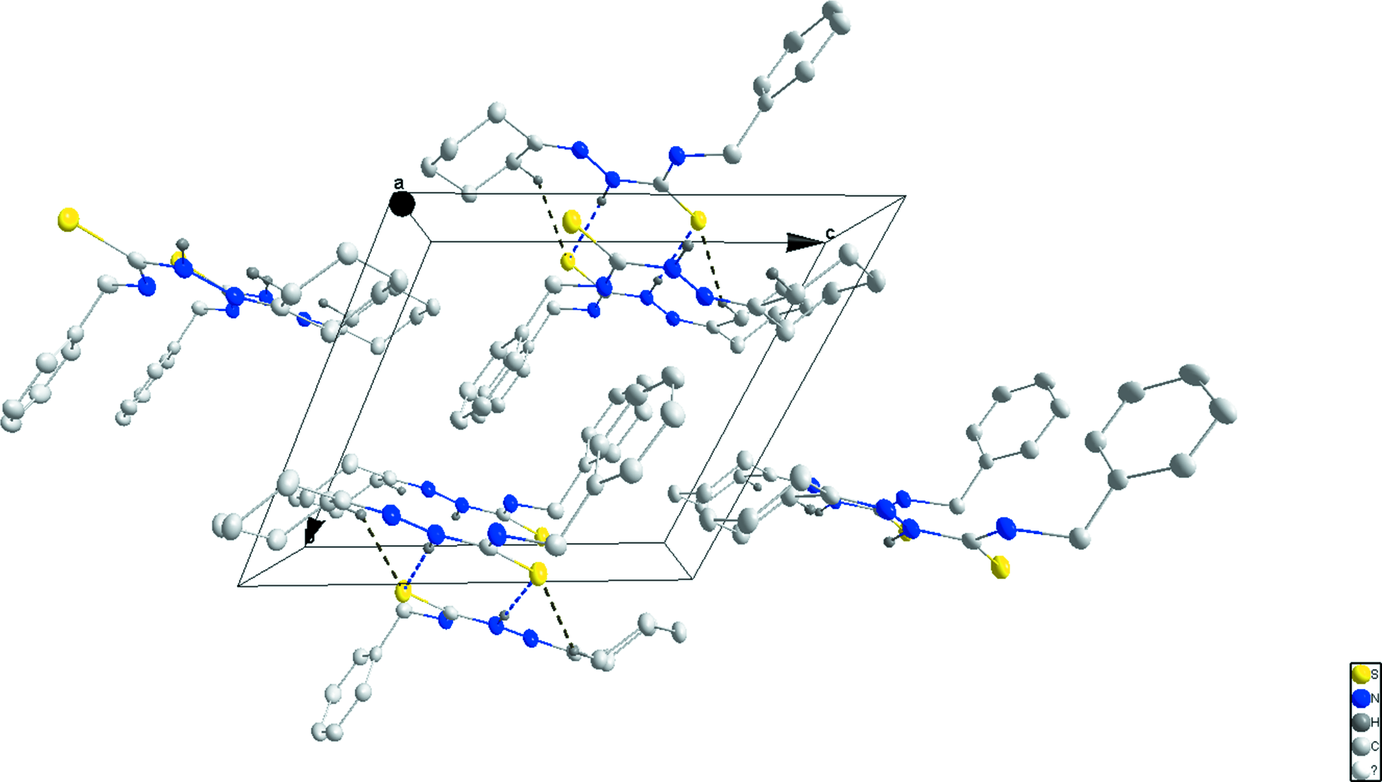

Supplement: Supplementary file 5 [file e-71-0o933-fig2.tif]
